# Evolutionarily Dynamic, but Robust, Targeting of Resistance Genes by the miR482/2118 Gene Family in the Solanaceae

**DOI:** 10.1093/gbe/evv225

**Published:** 2015-11-19

**Authors:** Sophie de Vries, Thorsten Kloesges, Laura E. Rose

**Affiliations:** ^1^Institute of Population Genetics, Heinrich-Heine University Duesseldorf, Germany; ^2^iGRAD-Plant Graduate School, Heinrich-Heine University Duesseldorf, Germany; ^3^Ceplas, Cluster of Excellence in Plant Sciences, Heinrich-Heine University Duesseldorf, Germany

**Keywords:** microRNA, gene family evolution, disease resistance, *R*-genes, miR482/2118, wild tomatoes

## Abstract

Plants are exposed to pathogens around the clock. A common resistance response in plants upon pathogen detection is localized cell death. Given the irreversible nature of this response, multiple layers of negative regulation are present to prevent the untimely or misexpression of resistance genes. One layer of negative regulation is provided by a recently discovered microRNA (miRNA) gene family, miR482/2118. This family targets the transcripts of resistance genes in plants. We investigated the evolutionary history and specificity of this miRNA gene family within the Solanaceae. This plant family includes many important crop species, providing a set of well-defined resistance gene repertoires. Across 14 species from the Solanaceae, we identified eight distinct miR482/2118 gene family members. Our studies show conservation of miRNA type and number in the group of wild tomatoes and, to a lesser extent, throughout the Solanaceae. The eight orthologous miRNA gene clusters evolved under different evolutionary constraints, allowing for individual subfunctionalization of the miRNAs. Despite differences in the predicted targeting behavior of each miRNA, the miRNA–*R*-gene network is robust due to its high degree of interconnectivity and redundant targeting. Our data suggest that the miR482/2118 gene family acts as an evolutionary buffer for *R-*gene sequence diversity.

## Introduction

Pathogens can exert strong natural selection on their hosts. Since potential pathogenic organisms are widespread in plant communities, plants require a fine-tuned and variable defense system. Essential components of the plant immune system are receptor proteins coupled with signaling proteins that detect pathogen molecules and subsequently mount a defense response. Of course, counter-adaptation by the pathogen leads to selection for pathogen expressed molecules that interfere with host detection or downregulate immune system genes.

A major class of R (resistance) proteins is represented by the nucleotide binding site-leucine rich repeats (NBS-LRRs; Meyers et al. 2005). These proteins recognize effectors in a direct or indirect fashion and are able to redirect the defense signaling and elicit *R-*gene-mediated immunity. In turn, NBS-LRR signaling can be undermined by pathogen effectors. Thus, the combination of a variable effector and *R-*gene complement is assumed to underlie the variety in host-pathogen specific interactions (Jones and Dangl 2006).

However, the presence or absence of these factors may not be the only determinant of specificity and orchestration of a timely defense response. Variation at the level of *R-*gene regulation may also contribute to the outcome of a host-parasite encounter. Proper regulation of *R-*genes can be important for the following reasons: 1) misregulation of *R-*genes can result in autoimmune responses and fitness costs in plants (Kim et al. 2010), 2) higher transcript abundance induced upon pathogen attack can lead to improved resistance (Cao et al. 2007; Bradeen et al. 2009; Kramer et al. 2009), and 3) faster transcriptional responses provide advantages in defense responses (Milling et al. 2011). Thus, exquisite and precise control of *R-*gene expression is no less important to the host or to the pathogen, although for opposing reasons.

Recently several microRNAs (miRNAs), 22 nt short small RNAs, have been predicted to target NBS-LRR encoding transcripts (e.g., Zhai et al. 2011; Li et al. 2012; Shivaprasad et al. 2012; Ma et al. 2014). miRNAs are encoded by so-called *MIR* genes. They are transcribed as primary miRNAs (pri-miRNA), which are further processed by a Dicer-like protein into a precursor miRNA (pre-miRNA). These pre-miRNAs are processed into the mature miRNA (Rogers and Chen 2013; Bologna and Voinnet 2014). In total, five different *MIR* gene families have been described to target domains in *NBS-LRR*s genes*.* One family, miR482/2118, stands out by having high sequence diversity among its family members (Shivaprasad et al. 2012). It consists of the two subfamilies, miR482 and miR2118, which both target the P-loop sequence motif in the *NBS-LRR* mRNA, and can be distinguished in the 5′-end of the mature miRNAs (Shivaprasad et al. 2012). The targeting of *NBS-LRR* mRNA leads to degradation of the *R-*gene transcript and production of phased secondary small interfering RNAs (phasiRNAs). The production of phasiRNAs strengthens the regulatory network by targeting the original and other *NBS-LRRs* for posttranscriptional regulation (Zhai et al. 2011; Shivaprasad et al. 2012).

Previous comparative small RNA transcriptome studies have described rapid evolution of miRNA families, including high birth and death rates via gene duplication, followed by functional redundancy, subfunctionalization, neofunctionalizaton, and pseudogenization (Maher et al. 2006; Nozawa et al. 2012). From genome-wide comparisons of *MIR* genes across several plant species, it has been found that the miRNA region and its complementary region (miR* region) show strong evolutionary constraints, while the surrounding pre-miRNA regions evolve more rapidly, with rates typical of intergenic regions (Fahlgren et al. 2010; Nozawa et al. 2012; Zhao et al. 2015). Most miRNAs target either a specific mRNA or small gene families. The evolution of miRNAs that target a large gene family, such as the *NBS-LRR* gene family, would potentially be exposed to different evolutionary constraints, than miRNAs with a more limited target repertoire.

The regulation of the miR482/2118 genes may be equally important as the regulation of *NBS-LRRs* themselves. Both transcriptional regulation of *MIR* genes and processing of pri-miRNAs to miRNAs can influence miRNA abundance. In plants and animals, nucleotide variation in the loop regions, but also in the 5′, 3′, and the miR/miR* complex, can influence these processes, leading to alternative miRNA sequences or differential miRNA expression (Krol et al. 2004; Liu et al. 2008; Todesco et al. 2012; Wang et al. 2013; Zhu et al. 2013).

In this study, we investigated the evolutionary history of the miR482/2118–*NBS-LRR* regulatory network in Solanaceae. We generated a data set of miR482/2118 genes from 14 species of Solanaceae and identified eight distinct gene types of this family. miRNA type and number are well-conserved in the group of wild tomatoes and, to a lesser extent, across the genus *Solanum.* However, all miRNA genes are predicted to be derived from duplication events predating the *Solanum*–*Nicotiana* split (∼24 Ma), with some even older than the Solanaceae itself, indicating subsequent remodeling of the miRNA repertoire after speciation. Comparisons of estimates of evolutionary rates among miRNA types show type-specific differences in evolutionary constraints: Some genes show the greatest sequence conservation in the miR and miR* regions, while others show the greatest sequence conservation in the 5′- and 3′-precursor regions. Target prediction analyses indicate a robust targeting network within and between species, due to a high target overlap between miR482/2118 genes. However, differential expression of miRNA in planta reveal that despite an overlap in potential targets, subfunctionalization may have played a role during the evolution of this gene family. Evolutionary robustness of this network (through redundancy of targeting) may help to support the rapid evolution of *NBS-LRRs* within tomato and potato.

## Materials and Methods

### Assembly of miR482/2118 Family Members from the Plant Kingdom

Sequences of the miR482/2118 genes annotated in miRBase v. 20 were downloaded (Griffiths-Jones 2004; Griffiths-Jones et al. 2006, 2008; Kozomara and Griffiths-Jones 2011). This included sequences from 29 plant species. The number of miR482/2118 family members from *Picea abies* was updated from four, reported in miRBase, to 24 recently reported by Xia et al. (2015). Additionally, we searched for these genes from the genomes of *Lotus japonicus* (Sato et al. 2008) and *Brachypodium distachyon* (International Brachypodium Initiative 2010) using nucleotide blast (BLASTn), because information in miRBase on these two species was sparse. All precursors and mature miRNA sequences of miR482/2118 from the Fabaceae and Poaceae available in miRBase v.20 were used as input for the searches. The draft genome of *Mimulus guttatus* (v. 1.0; Hellsten et al. 2013) was queried with the known precursor and mature miRNA sequences from *Solanum lycopersicum* published in Shivaprasad et al. (2012) and miRBase v. 20 to increase the representation of the Asterids in the analyses*.* To compare the gene family representation across the different species and to evaluate species-specific differences, we recorded the miR482/2118 gene numbers per species on the phylogeny. The phylogeny was based upon the following publications: Catalán et al. 1997; Yang et al. 1999; Wang et al. 2000; Wikström et al. 2001; Wojciechowski 2003; Swigoňová et al. 2004; Lavin et al. 2005; Delgado-Salinas et al. 2006; Van et al. 2008; International Brachypodium Initiative 2010; Pires and Dolan 2012.

### In Silico Identification of Additional miR482/2118 Precursor Sequences from the Solanaceae

To ensure that we had not overlooked or excluded any potential gene family members from our analyses, we searched the *S. lycopersicum* genome SL2.40 for matches to miR482/2118 sequences from *S. lycopersicum* and *Solanum tuberosum* identified from miRBase v. 20 and described in Shivaprasad et al. (2012; supplementary table S1, Supplementary Material online). This search was implemented using the PatMan-based sequence alignment tool implemented in the UEA small RNA workbench v 2.5.0 (Prüfer et al. 2008). Up to three mismatches were allowed per 22 nt-hit. We downloaded 450 bp upstream and 450 bp downstream of the predicted miRNA and performed structure predictions using the secondary structure tool of the CLC Main Workbench v. 6, sampling ten suboptimal structures each time. We subsequently shortened the approximately 900 bp to determine a good secondary structure of an appropriate length and then compared our results with the known pre-miR482/2118 sequences for *S. lycopersicum* and *S. tuberosum* from miRBase (supplementary fig. S4, Supplementary Material online). As a final step, we did a BLASTn querying all known/identified precursor miR482/2118 sequences of *S. lycopersicum* in the *S. tuberosum* genome and vice versa using Phytozome v. 9.1 (Goodstein et al. 2012) to ensure that we found the full set of miR482/2118 loci in both sequenced genomes.

To further extend the set of potential precursor sequences from the Solanaceae, we downloaded all miR482/2118 mature and precursor sequences from *Nicotiana tabacum* from miRBase v. 20*.* Using these sequences, we searched the following nucleotide and expressed sequence tag (EST) databases: *Nicotiana sylvestris* genome (Sierro et al. 2013), *Nicotiana benthamiana* leaf transcriptome (Bombarely et al. 2012), *Physalis peruviana* leaf transcriptome (Garzón-Martínez et al. 2012), *Capsicum annum* genome (Kim et al. 2014), *Solanum melongena* NCBI nucleotide databases, and the draft genome of *Solanum pimpinellifolium* (http://solgenomics.net/organism/Solanum_pimpinellifolium/genome [last accessed July 31, 2014] Ware et al. 2014; supplementary table S1, Supplementary Material online). Structure predictions were done as described above.

### Isolation of *MIR* Genes from *Solanum* Species

To increase the number of sequences from the Solanaceae, DNA was extracted from six species of the genus *Solanum*: *Solanum peruvianum* (LA1951 and LA2964)*, Solanum chilense* (LA3114)*, Solanum corneliomulleri* (LA1274)*, Solanum lycopersicoides* (LA2951), *Solanum ochranthum* (LA2682), and *S. melongena* (cv. Black Beauty). The plants were grown under standard greenhouse conditions prior to sampling. DNA extraction was performed as described in Edwards et al. (1991). Precursor sequences were amplified using polymerase chain reaction (PCR). PCR primers were designed to match regions surrounding the precursor sequences and showing high sequence conservation across *S. lycopersicum, S. pimpinellifolium*,** and *S. tuberosum.* Twenty microliters PCR reactions were conducted using 1:10 diluted *Solanum* DNA (50–100 ng), 1 × High Fidelity PCR Buffer, 2 mM MgSO_4_, 0.1 mM dNTPs, 0.2 µM each primer, and 0.05 U/µl Platinum *Taq* High Fidelity (Life Technologies). Primer sequences and annealing temperatures can be found in supplementary table S2, Supplementary Material online. Cycle conditions were according to the manufacturer’s instructions with 4 min initial denaturation phase and 7 min final extension. PCR products were purified using peqGOLD Cycle-Pure Kit (peqlab, VWR) according to the manufacturer’s instructions. PCR products were cloned using the TOPO TA Cloning Kit for subcloning with electro-competent One Shot TOP10 *Escherichia coli* cells (Life Technologies). Sequencing was performed by Eurofins MWG Operon (Germany). Sequences were aligned to the in vitro identified precursor sequences in MEGA 5.2.2 (Tamura et al. 2011) and quality was checked based on the sequence chromatograms. Secondary structure predictions were done as described above.

### Origin of the miR482/2118 Gene Family

**Fig. 1.—:**
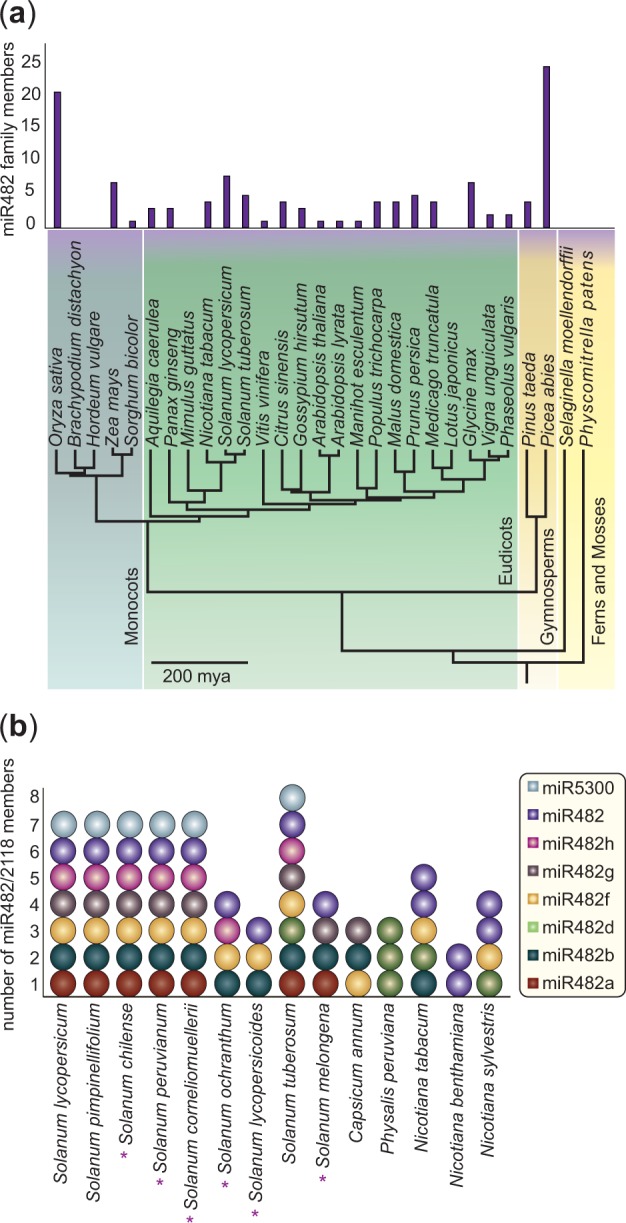
The repertoire of miR482/2118 in the plant kingdom. (*a*) Depicted are the number of miR482/2118 sequences for 29 plant species (based on literature, miRBase v. 20 and publically available genomes of *Mimulus guttatus, Lotus japonicas,* and *Brachypodium distachyon*) whose relationships are displayed in a literature-based phylogeny including all major land-plant lineages. (*b*) Presence and distribution of the miR482/2118 gene family across the Solanaceae. Purple asterisks indicate species in which some or all of the sequences were generated in this study.

*MIR* genes are proposed to originate along three different routes: from 1) protein-coding genes, 2) transposable elements (TE), or 3) *MIR* gene duplication. To determine which origin accounted for the diversification of the miR482/2118 family in the Solanaceae, we evaluated these three scenarios in turn. To find potential protein-coding sequences that may have resulted in the origin of the ancestral miR482/2118 genes, we conducted a BLASTn using all the genes in figure 1*b* against the NCBI EST, nucleotide and RefSeq databases. For evaluating the possibility of a TE derived ancestor, we used RepeatMasker Open-4.0 with the default settings for the internal libraries for Solanaceae (http://www.repeatmasker.org [last accessed August 4, 2014] Smit et al. 2014). To evaluate a large-scale duplication hypothesis for *MIR* genes, we concentrated on the *MIR* genes found in *S. lycopersicum* and followed the procedure described by Maher et al. (2006): we used a custom designed Perl script to extract the ten closest protein-coding genes on either side of each miR482/2118 precursor location from the *S. lycopersicum* genome. To identify potential paralogs we conducted a BLASTp with the ITAG2.4_proteins_full_desc.fasta from the *S. lycopersicum* SL2.50 release as our database and the extracted protein sequences as our query. Only matches of the query with proteins on chromosome 3, 4, and 6 were included in the further analysis. An e-value cutoff of less than 10e-04 was applied. We then mapped query versus database matches in order of their chromosome location to identify stretches of three or more conserved flanking genes, which would indicate large-scale duplication events. Tandem duplications are indicated by *MIR* genes that have the identical ten flanking protein-coding genes on both sides (Maher et al. 2006), that is, no protein-coding genes occur between the two *MIR* genes.

### Phylogenetic Analysis

We constructed a phylogeny to display the *MIR* gene duplication history using PHASE 3.0 (Jow et al. 2002; Allen and Whelan 2014). PHASE 3.0 is a phylogenetic program designed to study RNA evolution and was developed for RNA sequences that typically form secondary structures. Precursor sequences were aligned using ClustalW in MEGA 5.2.2 with the default settings (supplementary fig. S2, Supplementary Material online). The precursor sequences were approximately 130 bp long (range 84–171 bp). miR5300 sequences were excluded from the analysis because of their substantially longer precursor sequences (∼265 bp) and their partial switch of the 5′- and 3′-precursor arm. The gene Ghr-miR482a from cotton was chosen as an outgroup because its mature sequence (22 nt) is identical to sly-miR482f and it shares substantial similarity across the entire precursor with sly-miR482f (Shivaprasad et al. 2012). We performed model selection using a PHASE 3.0 implemented Perl script. The necessary secondary consensus structure of the precursor data set was created using a web-based version of RNAalifold (Bernhart et al. 2008; Gruber et al. 2008).

To infer the phylogeny, we applied a Markov–Chain–Monte–Carlo (MCMC) model based on two substitution models implemented in PHASE 3.0. Model 1 (REV + G with five gamma categories) is applied to unpaired bases of the predicted RNA structure. This substitution model is similar to those used for DNA sequences. Model 2 (RNA16D + G with five gamma categories) is applied to predicted paired regions in the secondary structure. Using two different substitution models for paired and unpaired regions, we can take into account the influence of secondary structure on the evolution of the molecule. We used 750,000 burn-in iterations and 1,500,000 sampling iterations with a sampling period of 150. We initiated the analysis with 11 different random seeds and afterwards computed the corresponding consensus trees using the mcmcsummarize function. All consensus trees resulted in the same clades with similar Bayesian probability support values. A subsequent analysis was conducted to investigate the paraphyletic relationship of the miR482 clade. A maximum likelihood tree based exclusively on the underlying nucleotide alignment and ignoring the secondary structure information was inferred assuming the K2 + G substitution model with five gamma categories.

### Estimating the Dates of miR482/2118 Duplication Events

To estimate the minimum age of the duplication events, we used MrBayes v 3.2.2 to determine whether the genes behaved according to a molecular clock (Ronquist et al. 2012). We assumed the 4by4 nucleotide model, mixed number of substitution types and gamma-distributed substitution rates with five categories. The strict molecular clock test was performed as described in the manual, however the number of generations was increased to 2,000,000. A molecular clock was rejected for the full data set; however, the removal of the miR482h clade, two *C. annum* sequences (Can-miR482b and Can-miR482g) and one *P. peruviana* sequence (Ppe-miR482c) resulted in a data set in which a strict molecular clock was the preferred model. Therefore, we inferred a phylogeny for this reduced data set. We estimated a rooted MCMC tree assuming a molecular clock in PHASE 3.0 based upon the previously described settings for the unrooted MCMC tree. We used the consensus tree output from the reduced data set as an input and allowed for no alterations in the phylogeny.

### Nucleotide Divergence in the miR482/2118 Family Members

We determined the average nucleotide divergence per site/per million years for the individual members in the miR482/2118 family in the Solanaceae. The sequences were first divided into precursor groups based on their clustering in the phylogeny. Then five precursor regions were determined within each precursor group: 5′-stem region, miR* region, loop region (between miR and miR*), miR region, and 3′-stem region. We determined the miR* region for each precursor sequence separately based on the precursor’s secondary structure. Each precursor group was aligned in ClustalW in MEGA 5.2.2 with the same settings as described above. Pairwise divergence between the precursor sequences within a precursor group was calculated. The number of fixed differences was estimated as described in Fahlgren et al. (2010). The number of fixed differences was divided by the length of the longer sequence in each pair. This was then divided by twice the divergence time in Myr, to calculate the substitution rate. These rates were averaged within a precursor group. For comparison, we did the same analysis on the miR166 family in the Solanaceae, collecting the known sequences deposited in miRBase.

### Target Prediction

To determine the potential targets of miR482/2118 miRNA genes in *S. lycopersicum* and *S. tuberosum,* we used the software psRNATarget: a plant small RNA target analysis server (Dai and Zhao 2011). As miRNA library input, all miR482/2118 from *S. lycopersicum* and *S. tuberosum* were used. As target library input, the coding sequences from *R-*genes annotated in *S. lycopersicum* and *S. tuberosum* were used (Lozano et al. 2012; Suresh et al. 2014). We conducted the analyses under two settings: a strict setting where the maximum expectation was set to ≤2.0 (i.e., low probability of false positives) and a less stringent setting where sequences with a score >2.0–3.0 were allowed.

### Target Homology and Network Visualization

The homology of the potential targets of miR482/2118 in *S. lycopersicum* and *S. tuberosum* to one another within and between species was determined using orthoMCL (Li et al. 2003; Fischer et al. 2011). The miRNA targeting and the evolutionary relationships of these putative targets were visualized in a network. The network was generated with Cytoscape 3.1.1 (Shannon et al. 2003). Evolutionary relationships between orthologous and paralogous target genes are displayed as undirected edges (i.e., lines). The predicted target-miRNA relationships are displayed as directed edges (i.e., arrows). Potential targets were grouped into gene families according to the predictions of orthoMCL. We determined gains and losses of potential miRNA targets in an evolutionary context. The network was clustered to see whether the miRNAs or the gene families are the major hubs of the network using the Fuzzifier clustering option in the clusterMaker package to allow nodes to belong to more than one cluster (Morris et al. 2011).

### Tissue-Specific Expression Patterns in *S. lycopersicum* and *S. pimpinellifolium*

We sampled roots and leaves of *S. lycopersicum* cv. M82 and *S. pimpinellifolium* LA114 to determine the expression of the mature miR482/2118 members in these plant tissues. Detection of the mature miRNA using quantitative PCR indicates that the miRNAs are present and correctly excised in the sample. These species can be grown axenically in the lab and both species are selfing, thereby avoiding issues caused by allele-specific differences in expression. The seeds were surface sterilized by treating them with 70% ethanol for 3 s, followed by 30 s approximately 5% sodium hypochlorite solution and then washed three times in sterilized water. Seeds were transferred on 1% H_2_O-agar, incubated in the dark at 18 °C to break the dormancy. Ten days postgermination (dpg) seedlings were transferred to 0.5% MS medium (Murashige and Skoog 1962), containing 1% sucrose. Samples were taken from 23 dpg old plants. We sampled four leaves from four different plants per biological replicate. The entire root (cut at the base of the hypocotyl) was sampled from four plants per biological replicate. Three biological replicates were conducted for the leaf sample and two biological replicates were conducted for the root sample.

RNA was extracted using the Gene Matrix Universal RNA/miRNA purification kit (Roboklon). cDNA was synthesized with the miScript II RT Kit (Qiagen) using the HiFlex buffer. The samples were diluted 1:100 and qPCR was performed with the miScript SYBR Green PCR Kit (Qiagen) in a 25 µl reaction in a CFX Connect Real-Time System (Bio Rad). Primers were designed based on the mature miR482/2118 sequences (supplementary table S2, Supplementary Material online). Three reference genes (TIP41, SAND, and AP2-complex subunit mu-1) were chosen based on Expósito-Rodríguez et al. (2008) and Dekkers et al. (2012). The reference gene primers were designed manually (for gene accessions and primer sequences see supplementary table S2, Supplementary Material online). The reaction protocol was used as specified for the miScript SYBR Green PCR Kit (Qiagen), with exception of the miR482a primer for which the annealing temperature was increased to 65 °C. The results were evaluated using the method described by Pfaffl (2001).

We tested whether the relative gene expression for each mature miRNA across the replicates was normally distributed. Differences in expression between mature miRNAs in the leaves versus roots of *S. pimpinellifolium* were analyzed using a two-tailed t-test with the assumption of unequal variances. A Mann–Whitney *U* test (Mann and Whitney 1947) was performed to determine whether mature miRNA transcript levels differed significantly between *S. lycopersicum* cv. M82 and *S. pimpinellifolium.*

## Results

### Distribution of miR482/2118 in Land Plants

To investigate the evolutionary history of the miR482/2118 family, we first determined the distribution of these genes in a subset of plant species based on publicly available data (see Materials and Methods, fig. 1*a*). The miR482/2118 family appears to be absent in mosses and Lycopods (*Physcomitrella patens* and *Sellaginella moellendorfii)* emerging first in the Gymnosperms, followed by an extensive radiation (fig. 1*a*). The gene family is also missing in some seed plants, although their close relatives possess several members (e.g., *M. guttatus* and *L. japonicus*; fig. 1*a*). In the species in which the gene family is present, it is typically represented by one to five members. The species *S. lycopersicum, Glycine max,* and *Zea mays* lie slightly above this with seven to eight family members. Certain species have many more members, such as *Pi. abies* with 24 and *Oryza sativa* with 21 family members (fig. 1*a*).

### Distribution of miR482/2118 in the Solanaceae

To gain insight into the more recent evolutionary history of the miR482/2118 family, we focused on plant species in the Solanaceae. To ensure that we had a complete repertoire of the miR482/2118 family in *S. lycopersicum* and *S. tuberosum,* we retrieved eight previously published mature miRNA sequences from *S. lycopersicum* and two miRNAs unique to *S. tuberosum* (Shivaprasad et al. 2012; miRBase v. 20)*.* Mapping these small reads to the *S. lycopersicum* reference genome resulted in 33 intergenic and 61 protein-coding sequences, which may be putative miRNA targets. Of the protein-coding sequences, 48 encode proteins having an NBS-LRR domain. Because none of the protein-coding sequences resulted in promising RNA secondary structures, they were not considered to be putative miRNA genes.

Of the 33 intergenic sequences, seven resulted in promising RNA secondary structures, one of which had not been previously identified in either of the two species, hereafter known as miR482h (supplementary fig. S1, Supplementary Material online). The sly-miR482c, sly-miR482d, sly-miR482e, and sly-miR482f genes mapped to the same region of the *S. lycopersicum* genome (Chr. 4, on position 55142317–55142338), with sly-miR482f showing no mismatches.

The BLASTn query of the precursor sequences yielded three additional genes (miR482f, miR5300, and miR482h) in *S. tuberosum,* but no genes in addition to those known from *S. lycopersicum.* No homologous sequences for stu-miR482b could be found in *S. lycopersicum*, not in the small read alignment, nor in the BLASTn query with the precursor sequences. Homologous sequences for sly-miR482, sly-miR482a, sly-miR482b, sly-miR482f, sly-miR482g, sly-miR482h, and sly-miR5300 could be found in *S. pimpinellifolium*, but not for stu-miR482b (fig. 1*b*). Analyses of members of the *Nicotiana* genus revealed five miR482/2118 members in *N. tabacum,* four members in *N. sylvestris,* and two members in *N. benthamiana* (fig. 1*b*)*.* EST, genome and transcriptome analyses resulted in one additional precursor sequences for *S. melongena,* three for *Capsicum annum* and three for *P. peruviana* (fig. 1*b*). Our PCR approach identified seven miR482/2118 family members for *S. peruvianum, S. chilense, S. corneliomulleri,* four members in *S. ochranthum,* three members in *S. lycopersicoides*,** and three members for *S. melongena.* The total miR482/2118 data set generated from the Solanaceae is comprised of 71 precursor sequences.

### The miR482/2118 Gene Family Diverged Prior to Speciation within the Genus *Solanum*

**Fig. 2.—:**
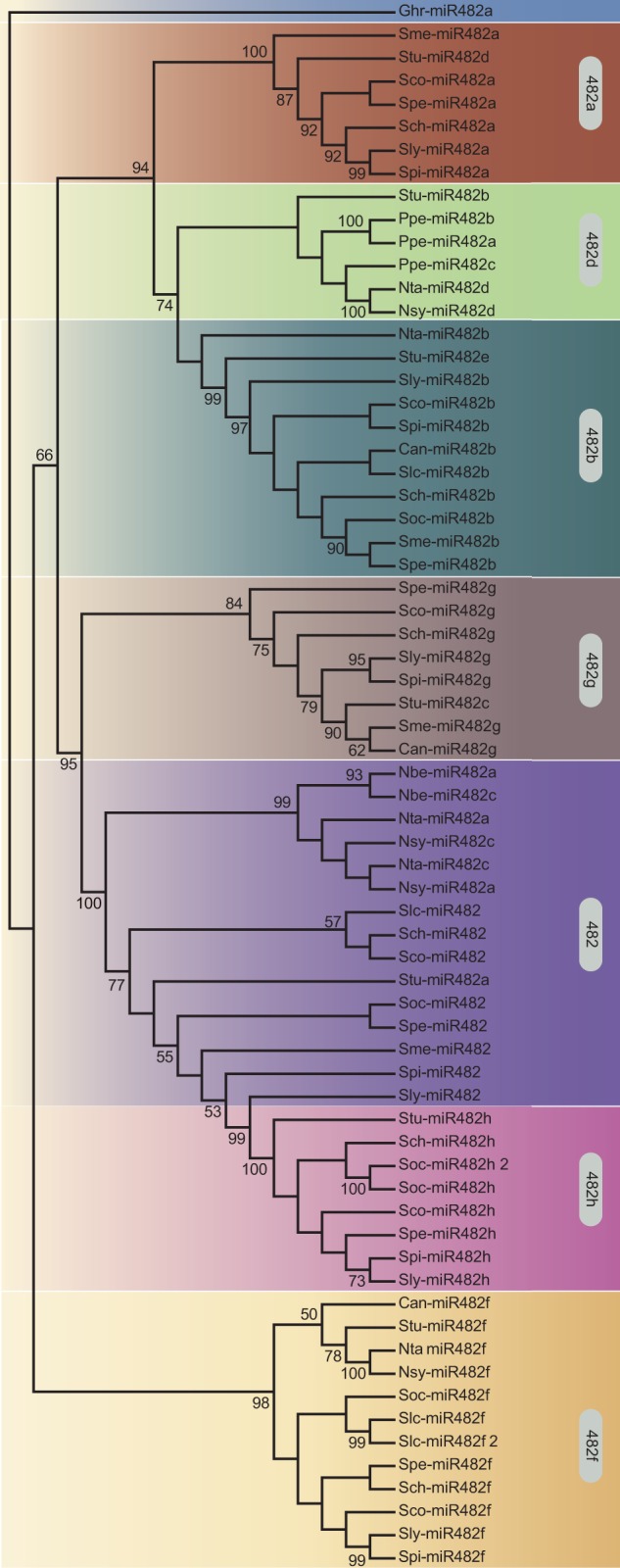
Cladogram of the miR482/2118 genes in the Solanaceae. The consensus Bayesian phylogeny of 67 miR482/2118 genes in seven miR482/2118 orthologous groups (distinguished by the different background colors). Bayesian posterior probabilities are indicated at the branches. Ghr, *Gossypium hirsutum* (outgroup); Sly, *Solanum lycopersicum*; Spi, *Solanum pimpinellifolium*; Spe, *Solanum peruvianum*; Sco, *Solanum corneliomulleri*; Sch, *Solanum chilense*; Soc, *Solanum ochranthum*; Slc, *Solanum lycopersicoides*; Stu, *Solanum tuberosum*; Sme, *Solanum melongena*; Can, *Capsicum annum*; Ppe, *Physalis peruviana*; Nta, *Nicotiana tabacum*; Nbe, *Nicotiana benthamiana*; Nsy, *Nicotiana sylvestris.* Bootstrap values greater than 50 are shown.

We reconstructed the evolutionary history of these genes from the Solanaceae using a Bayesian method assuming evolutionary models including both nucleotide substitution and RNA secondary structure. The individual genes form well-supported monophyletic clades, with the exception of one, miR482 (fig. 2). Because each clade includes genes from multiple species of Solanaceae, it is likely that the gene family diversified well before the divergence of these species.

The absence of reciprocal monophyly for miR482 and miR482h was further investigated. Reciprocal monophyly of these two genes is supported in an analysis based on sequence evolution, but excluding secondary structure (supplementary fig. S3*a*, Supplementary Material online). The secondary structure prediction of the miR482 gene from tomato and the miR482h gene from potato share high similarity, despite many differences in their underlying nucleotide sequences (supplementary fig. S3*b*, Supplementary Material online). We hypothesize that convergent evolution (or homoplasy) in this character confounds phylogenetic inference. The presence of these genes in tandem arrangement in syntenic positions in both plant genomes further supports the scenario that these two genes were present in the ancestor of tomato and potato (supplementary fig. S4, Supplementary Material online). Therefore, in view of the phylogenetic evidence and positional information, we can confidently assert that the miR482/2118 gene family diversified prior to speciation within *Solanum.*

**Fig. 3.—:**
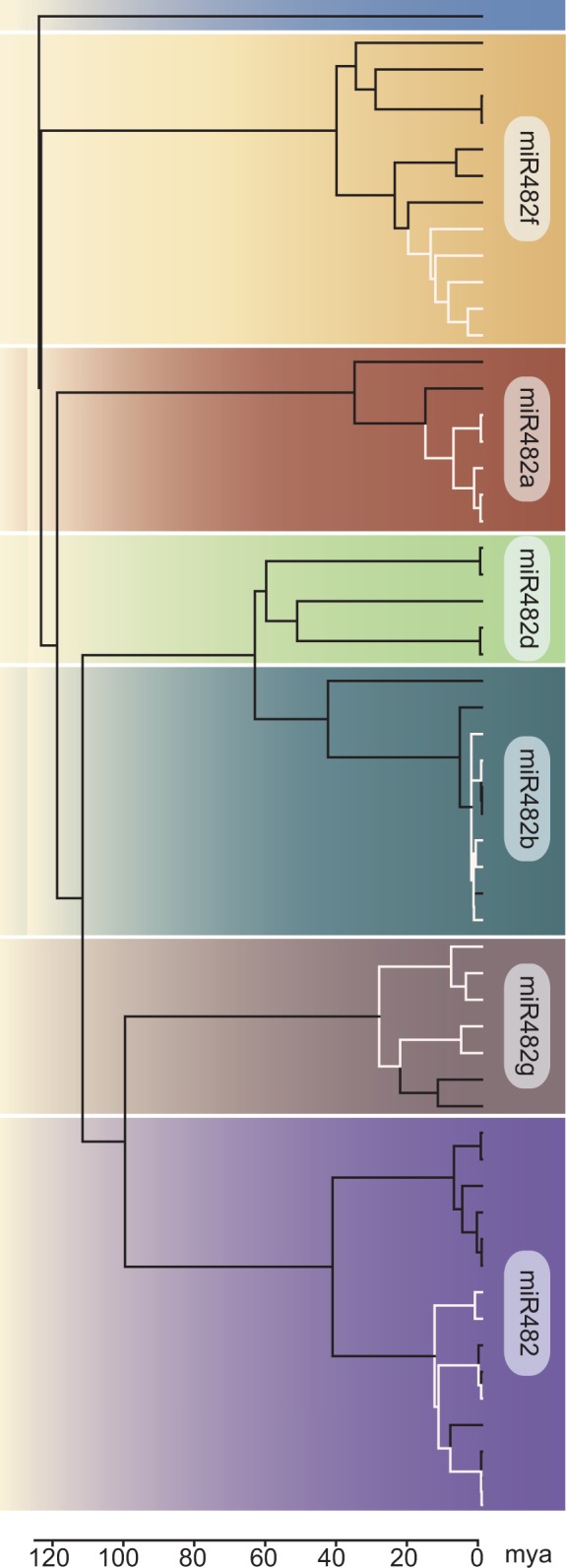
**Chronogram of the miR482/2118 genes.** A molecular clock was supported for a data set containing genes from six clades (distinguished by different background colors). The tree was calibrated by the rosid–asterid split (125 Ma). The miR482a gene from *Gossypium hirsutum* was used as an outgroup. The divergence times (in Ma) are above the line of the legend and the substitution rates are given below the line. The five species belonging to the wild tomato clade are indicated by white branches.

The chronogram also indicates that the miR482/2118 gene family expanded prior to speciation of the major crown lineages in the Solanaceae (fig. 3). The diversification of this family likely occurred more than 24 Ma (the age of the *Nicotiana–Solanum* split, Wu and Tanksley 2010; Särkinen et al. 2013). Only miR482h is found exclusively in tomatoes and potatoes, thus appearing to be of more recent evolutionary origin. In contrast, miR482d is found across several representatives of the Solanaceae, but is missing from the wild tomato clade (figs. 1*b* and 2). These patterns indicate that gene retention and, to some extent, gene loss has occurred within the Solanaceae.

We considered the three postulated origins for *MIR* genes: from 1) protein-coding genes, 2) TE, or 3) *MIR* gene duplication. No evidence for protein origin or TE transposition was found for the miRNA genes in *Solanum.* Maher et al. (2006) estimated the upper limit to detect historical duplication to be approximately 39 Ma. If these miRNA genes duplicated and diverged prior to the split of *Nicotiana* and *Solanum* (estimated to be ∼24 Ma), we may have some power to detect these ancestral duplications. However, because no trace in the regions surrounding the miR482/2118 loci in the *S. lycopersicum* genome can be found, the expansion of this gene family may have not only predated the split between *Nicotiana* and *Solanum,* but expanded even earlier. The colocalization of *MIR* genes in *S. lycopersicum* on chromosomes 3 and 6 (supplementary fig. S4, Supplementary Material online) together with the absence of coding genes located between these *MIR* genes is the only observation consistent with a history of recent tandem duplication.

The divergence time estimates shed light on the possible sequence of duplication events. Four members of the miR482/2118 gene family (miR482a, miR482b, miR482d, and miR482g) are located within 8.2 kb on chromosome 6. The most recent ancestral node shared by these four genes is 115 Ma (fig. 3). Successive rounds of duplication subsequent to this time point gave rise to these four genes, along with the other gene family members, miR482, miR482f, miR482h, and miR5300. Because miR482, miR482f, miR482h, and miR5300 are located on chromosomes other than chromosome 6, a translocation event, followed by the tandem duplication leading to miR482 and miR482h, must be postulated to explain the current chromosomal distribution of these gene family members.

### The miR482/2118 Network Shows Sequence Dependent Specialization and Evolutionary Robustness

Since the miR482/2118 gene family diversified prior to speciation of many taxa in the Solanaceae, we posed the question whether evolutionary patterns are conserved in miR482/2118 family members across species. We compared the evolutionary rates across miR482/2118 family members based on predefined structural regions of the predicted miRNA transcripts. These regions have been shown to be subject to different evolutionary constraints (Fahlgren et al. 2010). miR and miR* regions typically show the greatest constraint and hence the lowest rate of substitution, while the loop and stem regions, although important for processing, show lower constraint and hence a higher rate of substitution (Fahlgren et al. 2010). We analyzed the five regions of the miRNA genes (the miR region, the miR* region, the loop region, and the 3′- and 5′-stem regions) separately for each family member. We compared these rates to those estimated from the same plant species for the miR166 sequences. We chose the miR166 as a reference gene because it mirrored the typical substitution patterns of plant miRNAs described in Fahlgren et al. (2010), has a defined biological function and is wide-spread in the angiosperms.

**Fig. 4.—:**
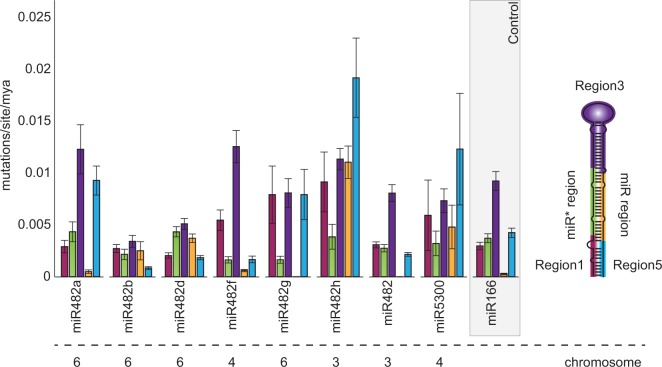
**Evolutionary rates of the miR482/2118 precursor sequences.** On the right, the canonical secondary structure of the miR482/2118 pre-miRNA is shown. Different coloring indicates the five functional regions of the pre-miRNA and is identical with the color coding of the bars in the histogram: 5′-region (red), miR* region (green), loop region (purple), miR region (yellow), and 3′-region (blue). miR166 is included for comparison. Below the graph, the chromosome for each gene is indicated.

Our data show that each miR482/2118 cluster has a unique evolutionary fingerprint (fig. 4). In the miR482b subclade, the stem and loop sequences are rather conserved, whereas the substitution rates in the miR and miR* regions were high. In contrast, the miR482g subclade showed little to no variation in the miR* and miR region, whereas stem and loop regions were less conserved (fig. 4). The rate of evolution of the miR region and 3′- and 5′-region were elevated in about half of the miR482/2118 clusters compared with other miRNA precursors, such as miR166 (fig. 4, Fahlgren et al. 2010). In general, at least one region had an elevated rate of substitution, except miR482.

We investigated whether these distinct evolutionary patterns correlated with a differences in target specificity across miR482/2118 genes. To do so, we predicted the targets of all miR482/2118 members in *S. lycopersicum* and *S. tuberosum* based on predicted *NBS-LRR* sequences. Although *S. tuberosum* possesses an additional miR482/2118 member, a similar proportion of *NBS-LRRs* are predicted to be targeted in both species: 19% (82 out of 434 *R*-genes) in *S. tuberosum* and 20% (52 out of 260 *R-*genes) in *S. lycopersicum* under the less stringent target prediction assumptions.

To identify general targeting patterns, we used orthoMCL to determine the *R-*gene families from the pool of predicted targets in *S. lycopersicum* and *S. tuberosum.* In general, the size of the *R-*gene family is positively correlated with the number of different miRNAs targeting the *R*-gene family (ρ = 0.69, *P* value = 0.012). In *S. lycopersicum* the predicted *R-*gene targets are more likely to be CC-NBS-LRRs, while in *S. tuberosum* the predicted *R-*gene targets are enriched for TIR-NBS-LRRs, suggesting differences in targeting between species (supplementary fig. S5, Supplementary Material online).

**Fig. 5.—:**
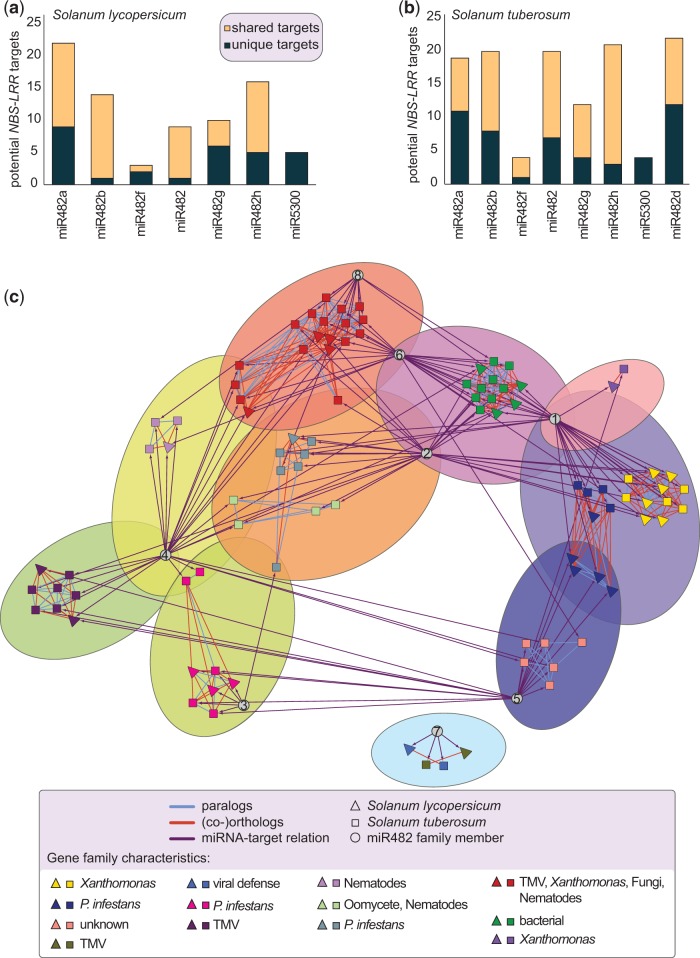
**Predicted *NBS-LRR* targets of the miR482/2118 family.** Unique *NBS-LRR* targets (dark turquoise) and targets shared between at least two paralogous miRNAs (yellow) in (*a*) *Solanum lycopersicum* and (*b*) *Solanum tuberosum.* (*c*) Network incorporating target predictions and evolutionary relationships between *NBS-LRRs* genes. OrthoMCL was used to determine gene families: genes from *S. lycopersicum* (triangles), genes from *S. tuberosum* (squares), orthologous relationships (orange lines), and paralogous relationships (light blue lines). Each gene family has a different color. The predicted miRNA-target relationships are indicated by direct edges (purple arrows). The miRNA nodes (gray circles) are numbered as follows: 1) miR482a; 2) miR482b; 3) miR482f; 4) miR482; 5) miR482g; 6) miR482h; 7) miR5300; and 8) miR482d. The nodes were clustered with Fuzzyfier according to connectivity, allowing shared clusters. Clusters are shown by the different background colors. 50% of miRNAs share clusters. Pathogen specificities of the different *R*-gene families are given in the legend.

The total number of potential targets and the degree of redundancy in targeting varies between the miR482/2118 members of *S. lycopersicum* and *S. tuberosum.* We observe a high number of *NBS-LRR* genes that are predicted to be targets of two or more different miR482/2118 family members (fig. 5*a* and *b*, supplementary fig. S6, Supplementary Material online). All miR482/2118 family members are predicted to possess unique targets and the number of unique targets increases with the stringency of the analysis (fig. 5*a* and *b*, supplementary fig. S6, Supplementary Material online). The structurally unique miR5300 has exclusive (i.e., unique) targets only under a less stringent target prediction, and no predicted targets under strict settings (fig. 5*a* and *b*, supplementary fig. S6, Supplementary Material online).

In *S. lycopersicum*, miR482a has a high number of targets, while miR482f has only a few. These two genes, although quite different in their predicted targeting behavior, do not differ greatly in their evolutionary rates across the five regions of the *MIR* gene and their miR regions show the greatest sequence conservation (fig. 4). In general, we found no correlation between the substitution rates in the miR region and the number of targets, suggesting that the number of targets is not the only determinant of the evolutionary trajectories among members of the miR482/2118 family.

Because *R-*genes differ in their pathogen specificity, we evaluated whether certain miR482/2118 members consistently targeted *R-*genes with similar pathogen specificities (i.e., is their evidence for subfunctionalization or specialization for *MIR* genes to control specific types of *R*-genes). We observed that individual miRNAs are predicted to target multiple *R*-genes with different pathogen specificities. Therefore we concluded that the miRNAs function mainly as generalists, rather than specialists. However, the degree to which the miRNAs are connected to any particular *R-*gene family varies. We predicted clusters in the network using Fuzzifier in clusterMaker to allow for nodes (e.g., miRNAs) to be shared between *R-*gene clusters. This resulted in ten clusters, made up of one to two *R-*gene families (fig. 5*c*). Half of the miR482/2118 genes are located at the intersection between two or more clusters. If we consider the pathogen specificity of the putatively targeted *R-*genes, we find that miR482f is typically associated with *Phytophthora infestans* defense responses, miR5300 with viral defense responses and miR482a with bacterial and *P. infestans* defense responses. The remaining miR482/2118 members show a more generalist’s pattern.

The observed target-dependent specialization of some miRNAs suggests that host-pathogen interactions do shape the evolution of these genes. To investigate potential evolutionary consequences of miRNA targeting for *R-*genes, we analyzed gains and losses within the predicted miRNA-target network (fig. 5*c* and supplementary fig. S7, Supplementary Material online). We observed that the paralogs within an *R-*gene family typically are predicted to be targeted by one or more of the same miRNA (86% in tomato and 80% for potato; supplementary fig. S7, Supplementary Material online). In contrast, orthologous *R-*genes (i.e., between species) are predicted to share one or more of the same miRNA only 63% of the time. Likewise, while greater than 30% of the paralogs are predicted to be targeted by exactly the same set of miRNAs, only 11% of the *R*-gene orthologs are predicted to be targeted by the exact same set of miRNAs. Therefore, targeting behavior is more strongly conserved between paralogs rather than between orthologs. Despite some conservation, 37% of orthologous *R-*genes between *S. lycopersicum* and *S. tuberosum* are predicted to be targeted by distinct miRNAs. This reflects the potential for divergent evolution in *R*-genes that can be matched by species-specific coevolution of miRNA genes over a relatively short time span (estimated split between tomato and potato ∼ 8 Ma).

### Differences in miR482/2118 Expression

**Fig. 6.—:**
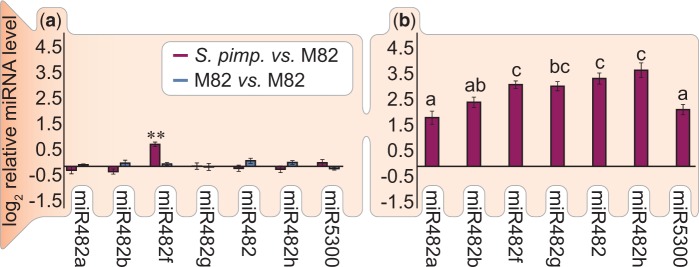
**Expression of miR482/2118 in *Solanum lycopersicum* and *Solanum pimpinellifolium*.** Expression levels of the miR482/2118 family members in *Solanum* species were determined using qPCR. (*a*) Expression of miR482/2118 in leaves of *Solanum pimpinellifolium* compared with *Solanum lycopersicum* cv*.* M82. (*b*) Comparison of expression of miR482/2118 in leaves versus roots of *S. pimpinellifolium.* Letters above the bars correspond to statistically distinct groups at *P* value < 0.05.

In addition to evaluating the evolutionary history and predicted targeting patterns, we tested if the mature miR482/2118 family members are expressed (i.e., implying correct excision from the precursor) and have species-specific or tissue-specific expression differences in the closely related species *S. pimpinellifolium* and *S. lycopersicum* using qPCR. For six mature miR482/2118 family members, no species-specific differences between *S. lycopersicum* and *S. pimpinellifolium* were detected (fig. 6*a*). However, miR482f is significantly upregulated in *S. pimpinellifolium* compared with *S. lycopersicum.*

In general, all mature miR482/2118 members have higher expression in leaves compared with roots (fig. 6*b*). Three statistically significantly different expression classes for these seven miRNAs could be detected: miR482, miR482f, and miR482g had the greatest upregulation, whereas miR5300 and miR482a had the least. The three miRNAs, miR482, miR482f, and miR482g, with the greatest upregulation share a number of predicted *R*-gene targets (figs. 5*c* and 6*b*). This indicates that expression patterns may correlate with the target profile. A surprising result was that miR482a and miR482g had different pattern of expression, but lie within 200 bp of one another in the genome. Differential transcript abundance may arise from differential processing of the pri- or pre-miRNAs. This physically closest pair of genes shows not only expression differences of the corresponding miRNAs, but also differences in targeting. This underscores the observation that despite redundancy in the predicted target network (i.e., targeting of the same *R*-gene by multiple miRNAs), some target specialization has arisen.

## Discussion

The miR482/2118 gene family is a negative regulator of plant resistance genes (Zhai et al. 2011; Li et al. 2012). It is an evolutionarily striking family due to its presence/absence variation throughout the plant kingdom and its high within and between species sequence variation of mature miRNA sequences. To better understand the evolutionary history of this gene family, we studied these genes in the Solanaceae with a focus on wild tomatoes.

### miR482/2118 Is an Ancient miRNA Family

Within the Solanaceae, we detected eight different genes belonging to the miR482/2118 gene family in the 14 species analyzed. In agreement with the study of Shivaprasad et al. (2012), we observed high variation in the mature miRNA sequences between species (fig. 4). However, the use of entire precursor sequences allowed us to identify orthologs between species, despite differences in their mature miRNA regions.

The miR482/2118 gene family repertoire of the nightshades diversified prior to speciation within the Solanaceae. Some duplication events appear to have occurred more than 100 Ma, corresponding to the split between the Phrymaceae, the family to which *Mimulus* belongs, and the Solanaceae. While sequences related to miR482/2118 can be found in the *M. guttatus* genome, they do not appear to fold, indicating subsequent gene loss or degeneration in this species.

Although it was not possible to reconstruct the ancient large-scale duplication events, we could identify younger tandem duplications between miR482a, miR482b, miR482d, and miR482g on chromosome 6 and miR482 and miR482h on chromosome 3. Based on our observations, we hypothesize that miR482/2118 is an ancient miRNA family that radiated early on in plant evolution involving large-scale and tandem duplication events. The observed variation in the mature miRNA regions of orthologous miR482/2118 genes between even closely related species (e.g., within *Solanum*) is best explained by species-specific miR482/2118 repertoire and its coevolution with its targets, rather than convergent evolution leading to a unique miR482/2118 inventory in each species, as previously suggested (Shivaprasad et al. 2012). A recent study of this gene family in the gymnosperm species, *Pi. abies*, indicates that the gene family originated following the split between ferns and Gymnosperms (Xia et al. 2015).

### miR482/2118 Homologs Show Unique Evolutionary Fingerprints

*R*-genes are typically dynamic and fast evolving components of plant genomes (Clark et al. 2007). However, within a given *R*-gene family, evolutionary rates can differ among paralogs, with some showing rapid evolution and others evolving more slowly (Kuang et al. 2004). We see an analogous situation for the miR482/2118 family in the Solanaceae.

Evolutionary rates are predicted to be lower for taxonomically widespread miRNAs maintained over long evolutionary timescales (so-called ancient or conserved miRNAs) than for miRNAs of more recent origin (Fahlgren et al. 2010; Meunier et al. 2013). miR166 is one such conserved miRNA and helps to control root development (Carlsbecker et al. 2010; Fahlgren et al. 2010). In contrast, the miR482/2118 family, although widespread and ancient (Xia et al. 2015), departs from this pattern. Members of this gene family show an elevated rate in at least one region of the precursor sequence. Coevolution with their *R*-gene targets, which themselves show elevated sequence evolution, may contribute to differences in evolutionary history compared with more conserved miRNAs.

### miR482/2118 Targeting Is Highly Dynamic and May Function as a Buffer in R-Gene Evolution

miR482/2118 family members are predicted to target a large subset of *R*-genes. Despite their differences in evolutionary rates, these miRNAs appear to be mainly generalists based on the pathogen spectrum of their potential *R*-genes targets. To investigate this paradox, we explored the predicted *R*-gene–miRNA targeting relationships in more detail. We observed that despite several unique predicted targets, nearly all miRNA genes share some predicted targets. This results in a highly interconnected network, in which larger *R*-gene families are predicted to be targeted by more miRNAs compared with smaller *R-*gene families. A correlation between *R-*gene family size and number of targeting miRNAs has been observed in other species (González et al. 2015). Hence, this robustness seems to be conserved in evolution and is potentially an important feature of *R-*gene regulation.

Fei et al. (2013) put forth the hypothesis that miRNAs act as buffers for *R-*genes. They argued that multiple layers of regulation may be evolutionarily beneficial since mutations introduced into *R*-gene promoters could lead to autoimmune responses and thus high fitness costs for the plant. Another hypothesis put forward by Li et al. (2012) and Shivaprasad et al. (2012) is that *R*-gene targeting by small RNAs help to support *R*-gene evolution. They suggest that transcriptional suppression could dampen fitness costs of poorly functioning *R-*genes and thus relax constraints on *R-*gene sequences; ultimately leading to *R-*gene diversification, sub- and neofunctionalization. These two hypotheses are not mutually exclusive, and in fact, can be united. We show that *R-*genes in *S. lycopersicum* and *S. tuberosum* are typically predicted to be targeted by two or more miR482/2118 members. This redundancy likely protects against the negative consequences of misexpression of *R-*genes. It may also allow for cryptic genetic variation to accumulate at *R-*genes.

We also observed that fewer orthologous *R*-genes between *S. lycopersicum* and *S. tuberosum* are predicted to be targeted by the same miRNA compared with paralogs within species. This may arise from species-specific *R-*gene duplications and divergence subsequent to speciation (i.e., independent diversification of *R*-gene families following speciation). Therefore, divergence in targeting may have been concomitant with changes in *R-*gene repertoire after these species split. This may be advantageous to continue to maintain the appropriate suppression of targets. All in all, this points to a high buffering capacity of the targeting network, in which potential losses of targeting relationships are off-set by the high interconnectivity of the network, despite ongoing subfunctionalization of the miRNAs.

## Supplementary Material

Supplementary figures S1–S7 and tables S1 and S2 are available at *Genome Biology and Evolution* online (http://www.gbe.oxfordjournals.org/).

Supplementary Data
