# Supplementary material for: Evolutionarily Dynamic, but Robust, Targeting of Resistance Genes by the miR482/2118 Gene Family in the Solanaceae
Source: Genome Biol Evol. 2015 Nov 19;7(12):3307–21. doi: 10.1093/gbe/evv225 (PMC4700956; doi:10.1093/gbe/evv225)
Supplement: Supplementary Data [file supp_7_12_3307__index.html]

Evolutionarily Dynamic, but Robust, Targeting of Resistance Genes by the miR482/2118 Gene Family in the Solanaceae — Supplementary Data 

# Evolutionarily Dynamic, but Robust, Targeting of Resistance Genes by the miR482/2118 Gene Family in the Solanaceae

## Supplementary Data

files

- Supplementary Data - zip file
