# Supplementary material for: Evolutionarily Dynamic, but Robust, Targeting of Resistance Genes by the miR482/2118 Gene Family in the Solanaceae
Source: Genome Biol Evol. 2015 Nov 19;7(12):3307–21. doi: 10.1093/gbe/evv225 (PMC4700956; doi:10.1093/gbe/evv225)
Supplement: Supplementary Data [file supp_evv225_suppl_data.zip › TableS2.docx]

Table S2 Primer sequences

| **Name** | **Forward 5’-3’** | **Reverse 5’-3’** | **Annealing** | **Task** |
| --- | --- | --- | --- | --- |
| miR482a | GTTGCACTATATAATGAACAC | CATGACAATCTCTAAATCTCA | 56°C | Amplification/  Identification |
| miR482b | TCCATGAATGATACAAGAAA | GATGCAACAGATTCAACAA | 52°C | Amplification/  Identification |
| miR482f | TGAAAGGGATGAGAGTATTG | ATGAAGAAAGGGAGAAGCTA | 56°C | Amplification/  Identification |
| miR482d | GTACAATGAGTAAGATGAAG | AGAGCATACCAAGAAACCT | 54°C | Amplification/  Identification |
| miR482d R2 | - | AGAAACCTATTATGCAACTG | 54°C | Amplification/  Identification |
| miR482g | CTCGTACCCATTTTGTTTCT | TATGCAATACCAAAAGCAGA | 54.5°C | Amplification/  Identification |
| miR482 | AGGTTCAGTTTAGCAGAT | TGATCGATCGATAGCATT | 52°C | Amplification/  Identification |
| miR482h | TAATAAAGGCTTCCACAGCTAA | AATCTCGATGAATAATAGATA | 52°C | Amplification/  Identification |
| miR5300 | TATGATTCACCCTTATTTATG | CATTAGTAAACACAAGATTGT | 54.5°C | Amplification/  Identification |
| miR5300 R2 | - | ACACAAGATTGTGCCATATCT | 56°C | Amplification/  Identification *Solanum chilense* |
| miR482a | TCTTGCCTACACCGCCCATGCC | - (supplied in kit) | 65°C | qPCR |
| miR482b | TCTTGCCAATACCGCCCATTCC | - (supplied in kit) | 55°C | qPCR |
| miR482f | TCTTTCCTACTCCTCCCATACC | - (supplied in kit) | 55°C | qPCR |
| miR482g | TTTCCTATTCCACCCATGCCAA | - (supplied in kit) | 55°C | qPCR |
| miR482 | TTTCCAATTCCACCCATTCCTA | - (supplied in kit) | 55°C | qPCR |
| miR482h | TTACCAATTCCACCCATTCCTA | - (supplied in kit) | 55°C | qPCR |
| Sly-miR5300 | TCCCCAGTCCAGGCATTCCAAC | - (supplied in kit) | 55°C | qPCR |
| Spi-miR5300 | TCTCCAGTCCAGGCATTCCAAC | - (supplied in kit) | 55°C | qPCR |
| AP-2 complex subunit mu-1(Solyc08g006960.2) | AGTTTGTTGTTGAGGCTGTTA | CATAATTTCATCCAATAGCTC | 55°C | qPCR (Reference gene) |
| SAND (SGN-U316474) | GGCAATGAGGATGATGCTTC | TCATCTCCATATCTCGAGTA | 55°C | qPCR (Reference gene) |
| TIP41 (SGN-U321250) | TCTGGCTTAGGGTTGATGGA | GGGATATCCTTTCGAAGACA | 55°C | qPCR (Reference gene) |
| Nta-482d_inner | AGTCGGGAAGATGCCA | CGTGGGAAGATTTCATCT | 52°C | Amplification/Identification |
| Stu-miR482b | GTACAATGAGTAAGATGAAG | AATCAGAGCTATTACACAT (xxx) | 54°C | Amplification/Identification |
| Stu-miR482b_R2 | - | AGAAACCTATTATGCAACTG | 54°C | Amplification/Identification |
