# Supplementary material for: Evolutionarily Dynamic, but Robust, Targeting of Resistance Genes by the miR482/2118 Gene Family in the Solanaceae
Source: Genome Biol Evol. 2015 Nov 19;7(12):3307–21. doi: 10.1093/gbe/evv225 (PMC4700956; doi:10.1093/gbe/evv225)
Supplement: Supplementary Data [file supp_evv225_suppl_data.zip › Supplementary_Figures.pdf]

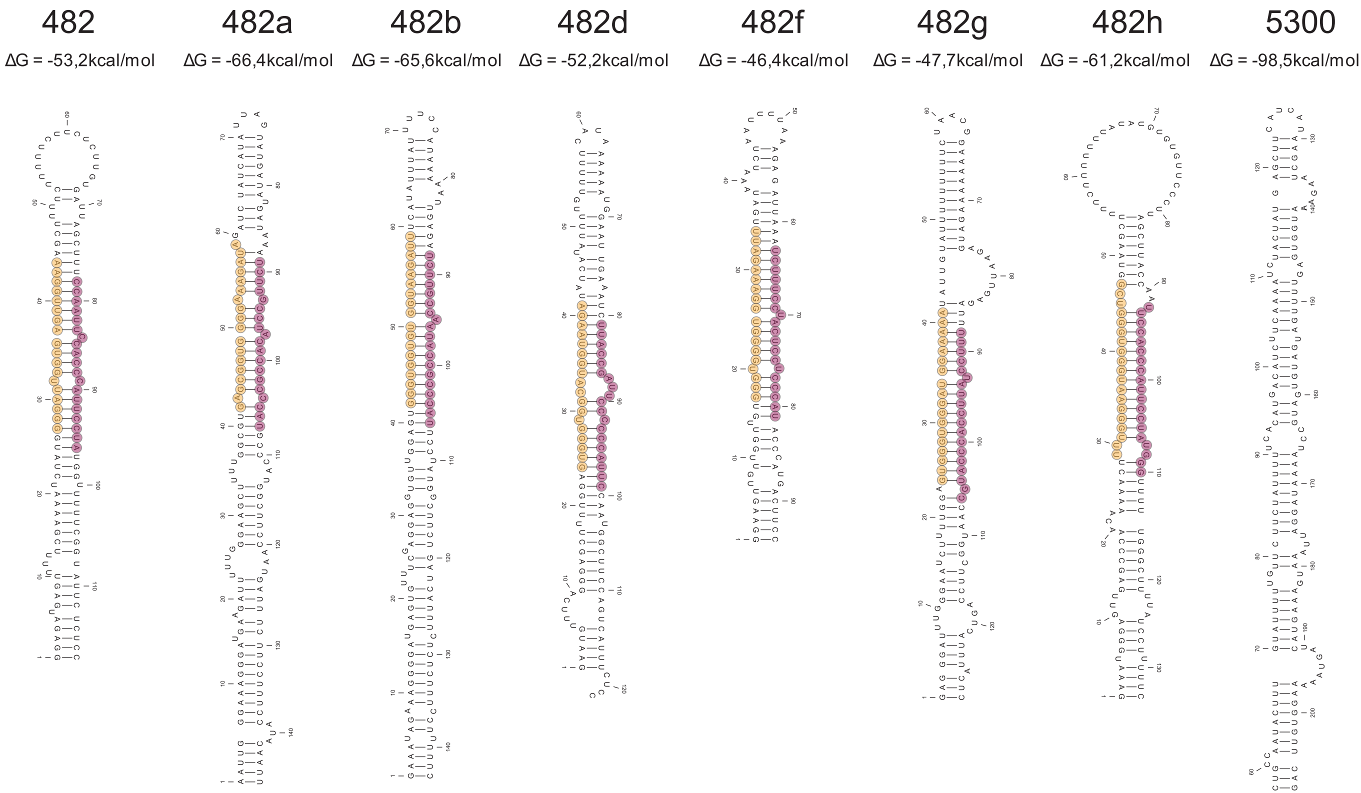

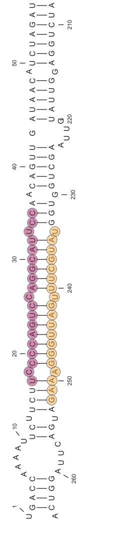

Figure S1 Predicted RNA secondary structures of the miR482/2118 genes

All miR482/2118 family members are predicted to form energetically stable secondary RNA structures (ΔG values). Shown here are the secondary structures of the *S. lycopersicum* sequences, with the exception of miR482d, which is not present in the *S. lycopersicum* genome. For miR482d, the predicted fold for *S. tuberosum* is shown. The miR region (purple) is on the 3' arm of the miRNA precursor. Only in the exceptionally longer precursor sequence of miR5300 are the miR (purple) and miR* region (yellow) swapped.

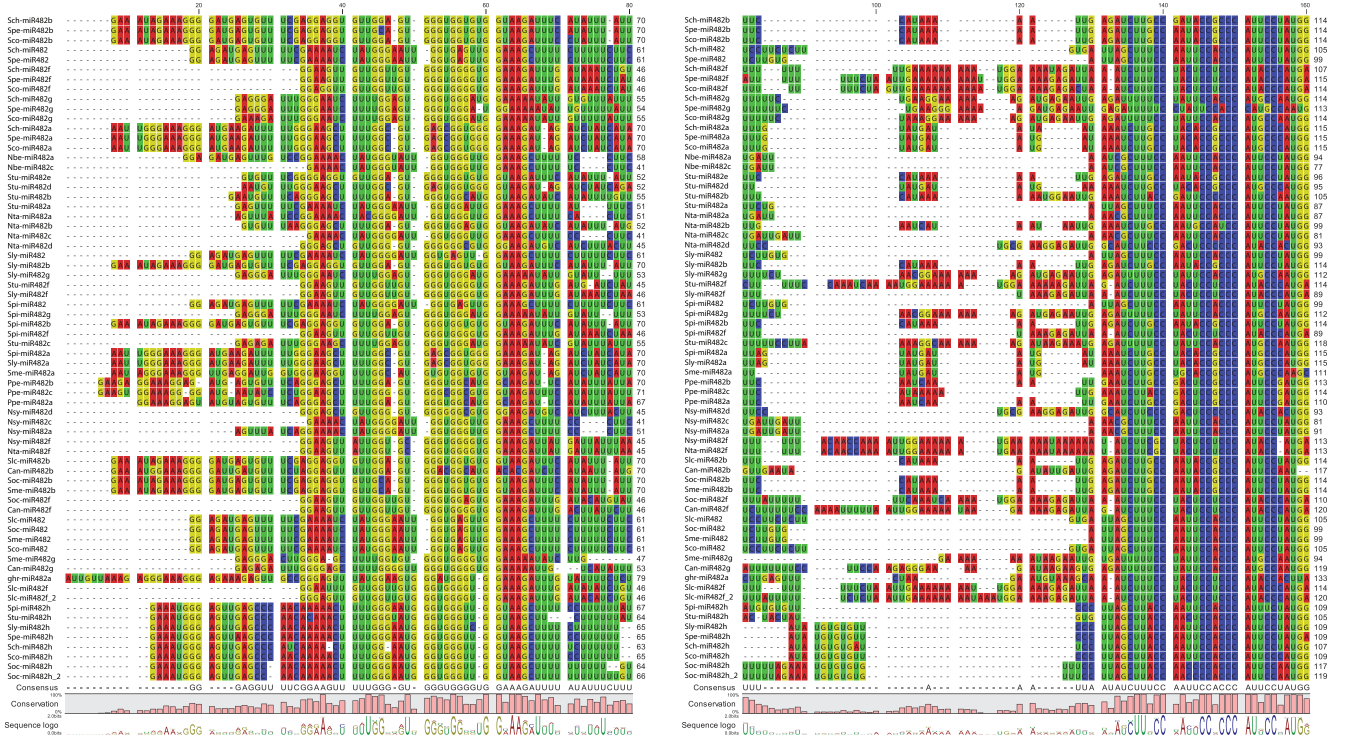

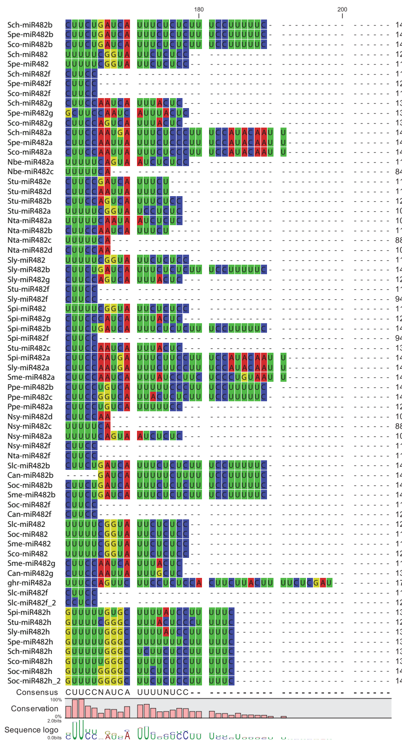

Figure S2 Alignment of pre-miR482/2118 sequences

The alignment (performed with Clustal-W) of the 68 miR482/2118 precursor sequences. Below the alignment is the 100% consensus sequence, histogram of conservation and sequence logo.

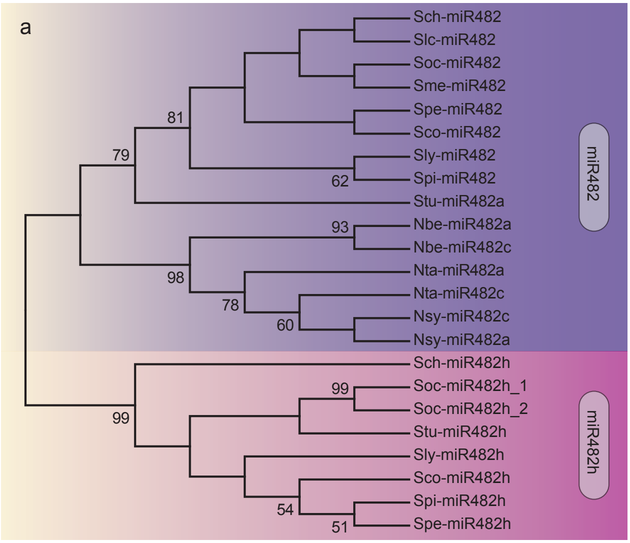

Figure S3 Phylogenetic analyses of pre-miR482 and pre-miR482h

(a) The miR482/miR482h cladogram. The numbers represent the bootstrap support of the nodes. Values below 50 are not shown. (b) Alignment of the precursor sequences of miR482 and miR482h. The consensus sequence, sequence conservation (0 - 100%) and the sequence logo (0 - 2 bits) are shown below the alignment.

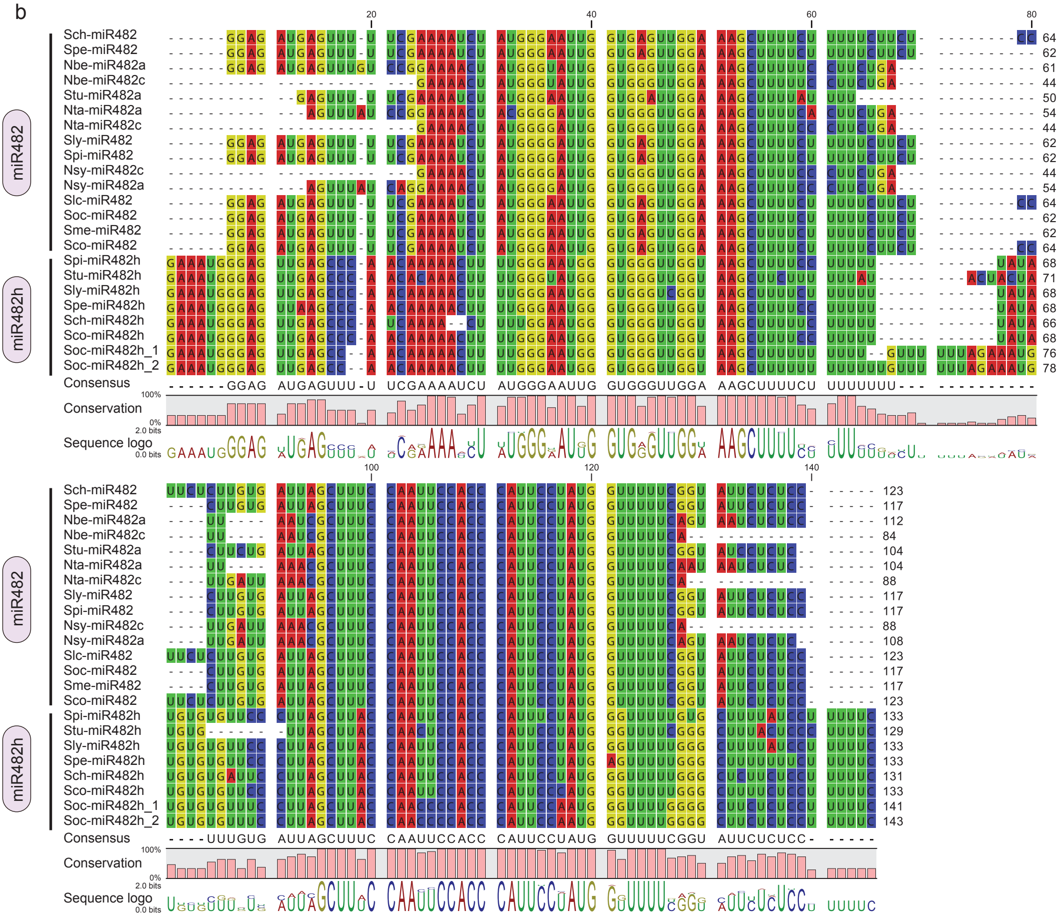

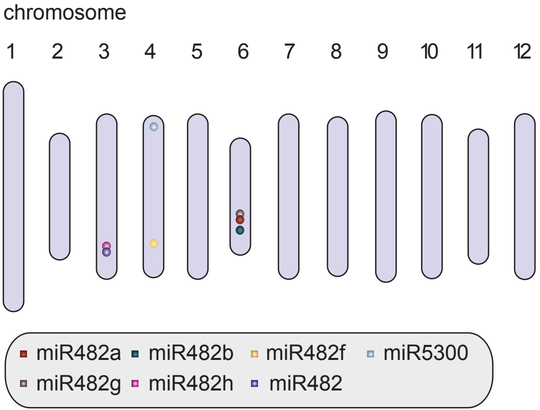

Figure S4 schematic chromosomal location of the miR482/2118 family members in *Solanum lycopersicum*
The seven miR482/2118 members of *S. lycopersicum* are distributed over chromosomes 3, 4 and 6. Five of the seven miRNAs are located in clusters. Cluster 1 is on chromosome 3: miR482 (dark purple) and miR482h (pink) are located 2999 bp apart. Cluster 2 is on chromosome 6: miR482a (red), miR482g (light purple) and miR482b (green) are less than 10kb apart.

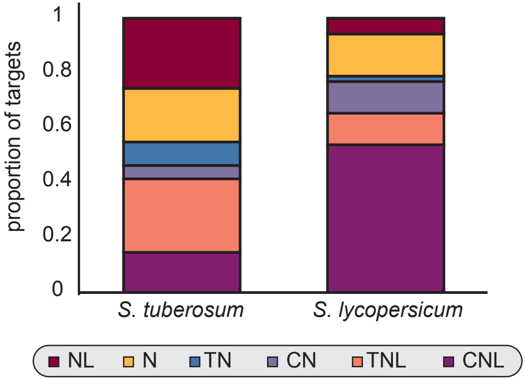

Figure S5 Distribution of the different NBS-LRR types as targets

NBS-LRRs are categorized into six different types: coiled-coiled-NBS-LRRs (CNL), Toll-interleukine-like-NBS-LRRs (TNL), those that lack the LRR domain (CN and TN), those that lack the coiled-coiled or Toll-interleukine-like domains (NL), and those that have only the NBS domain (N). *S. tuberosum* is enriched for the TNL type in its miR482/2118 targets, while *S. lycopersicum* is enriched for the CNL type. This is consistent with the NBS-LRR distribution in the two plant genomes.

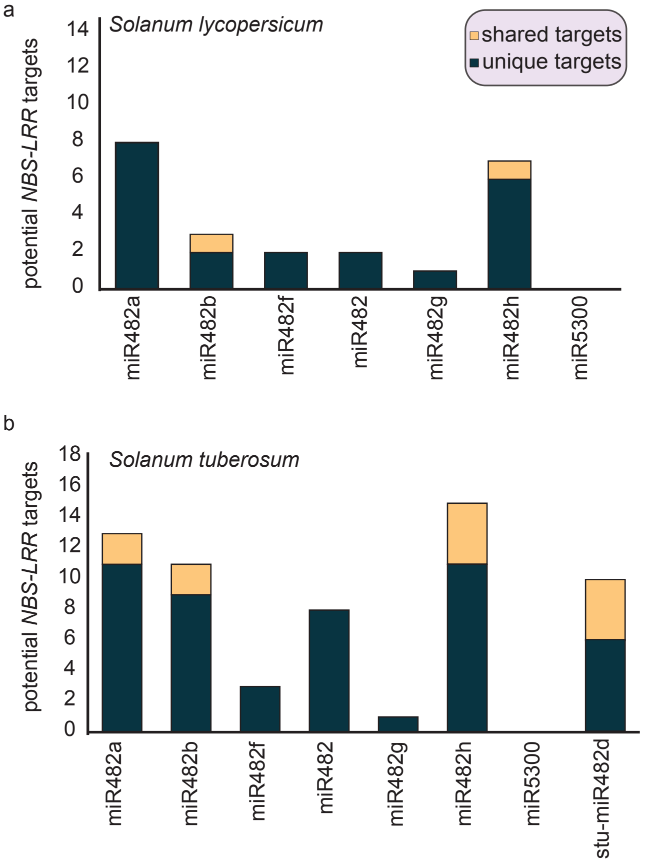

Figure S6 Number of *NBS-LRR* targets of the miRNA family members in *Solanum lycopersicum* and *Solanum tuberosum*

The distribution of target type (shared or unique) under the strict prediction settings. Compared to less stringent setting, many shared targets (yellow) are lost. The set of targets is exclusively unique (dark green) for miR482, miR482f and miR482g in both species and miR482a in *S. lycopersicum*. miR5300 loses all of its predicted targets in both species under the strict settings.

Case	i) exactly the same miRNAs	ii) some shared miRNAs	iii) no shared miRNAs
	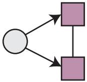	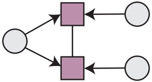	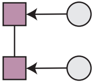
orthologs	*11%*	*51%*	*37%*
paralogs in *S. lycopersicum*	*34%*	*46%*	*20%*
paralogs in *S. tuberosum*	*31%*	*55%*	*14%*

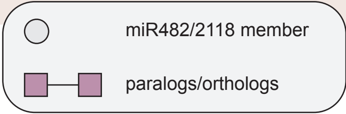

Figure S7 Gains and losses in miR482/2118 targeting

Analysis of gains and losses of *R* gene targets during gene family evolution within species (paralogs) or between species (orthologs). Three scenarios were identified: i) the orthologous miRNAs of *Solanum lycopersicum* and *Solanum tuberosum* retain their targets after target duplication, ii) retention of some but not all targets or iii) orthologous/paralogous targets are targeted only by different miR482/2118 members.
