# Supplementary material for: Evolutionarily Dynamic, but Robust, Targeting of Resistance Genes by the miR482/2118 Gene Family in the Solanaceae
Source: Genome Biol Evol. 2015 Nov 19;7(12):3307–21. doi: 10.1093/gbe/evv225 (PMC4700956; doi:10.1093/gbe/evv225)
Supplement: Supplementary Data [file supp_evv225_suppl_data.zip › TableS1.docx]

**Table S1** Accession and/or genome locations of miR482/2118 precursor sequences

| Plant species | 482 | 482a | 482b | 482d | 482f | 482g | 482h | mir5300 |
| --- | --- | --- | --- | --- | --- | --- | --- | --- |
| *Solanum lycopersicum* cv. Heinz1706 | miRBase MI0018482 | miRBase MI0020250 | miRBase MI0020251 | nd | **Genome chr4:55142255-55142348** | **Genome chr6:33869902-33870030** | **Genome chr3:58816161-58816293** | miRBase MI0018477 |
| *Solanum pimpinellifolium* LA114 | **draft genome contig 6606463** | **draft genome contig 27964** | **draft genome contig 6575598** | nd | **draft genome contig 436079** | **draft genome contig 27964** | **draft genome contig 6626949** | **draft genome contig 3698343** |
| *Solanum chiliense* LA 3114 | **Cloned**  **KP665226** | **Cloned**  **KP665232** | **Cloned**  **KP665238** | nd | **Cloned**  **KP665244** | **Cloned**  **KP665247** | **Cloned**  **KP665251** | **Cloned**  **KP665255** |
| *Solanum peruvianum* LA2964(miR482a) / LA 1951(all others) | **Cloned**  **KP665227** | **Cloned**  **KP665233** | **Cloned**  **KP665239** | nd | **Cloned**  **KP665245** | **Cloned**  **KP665248** | **Cloned**  **KP665250** | **Cloned**  **KP665256** |
| *Solanum corneliomuellerii*  LA1274 | **Cloned**  **KP665228** | **Cloned**  **KP665234** | **Cloned**  **KP665240** | nd | **Cloned**  **KP665246** | **Cloned**  **KP665249** | **Cloned**  **KP665252** | **Cloned**  **KP665257** |
| *Solanum lycopersicoides* LA2951 | **Cloned**  **KP665229** | nd | **Cloned**  **KP665235** | nd | **Cloned**  **KP665242; KP665243** | nd | nd | nd |
| *Solanum ochranthum* LA2682 | **Cloned**  **KP665230** | nd | **Cloned**  **KP665236** | nd | **Cloned**  **KP665241** | nd | **Cloned**  **KP665253**  **KP665254** | nd |
| *Solanum tuberosum* | miRBase MI0020245 | miRBase MI0020248 | miRBase MI0020249 | miRBase MI0020245 | **Genome chr4:50305049-50305167** | miRBase MI0020244 | **Genome chr3:40639562-40639690** | **Genome chr4:263855-264118** |
| *Solanum melongena* | **Cloned**  **KP665231** | **FS019499.1** | **Cloned**  **KP665237** | nd | nd | **FS019500.1** | nd | nd |
| *Capsicum annum* cv. CM334 | nd | nd | **KI878449** | nd | **GD100180.1** | **KI878449** | nd | nd |
| *Physalis peruvianum* | nd | nd | nd | **SRR111938.319534, SRR111938.200069,**  **SRR111938.133379** | nd | nd | nd | nd |
| *Nicotiana tabacum* | MI0020247,  MI0021417 | nd | miRBase MI0020252 | miRBase MI0021466 | **BP533636.1** | nd | nd | nd |
| *Nicotiana benthamiana* | **draft genome Niben044Scf00034318Ctg003*** | nd | nd | nd | nd | nd | nd | nd |
| *Nicotiana sylvestris* | **gi\|500491528:2301-3500*** | nd | nd | **gi\|492171585:1501-2700** | **gi\|500586900:4401-5500** | nd | nd | nd |

In bold are those pre-miRNA sequences that we identified within this study as pre-miR482/2118 sequences; * possibly form two splice variants based on the two precursor variants known from *Nicotiana tabacum*
